# A Case of Severe Hemorrhagic Cystitis Caused by Melphalan with Successful Bladder Preservation by Ligation of Bilateral Internal Iliac Arteries

**DOI:** 10.1155/2010/569138

**Published:** 2010-06-14

**Authors:** Takumi Takeuchi, Masayoshi Zaitsu, Koji Mikami, Yuta Takeshima, Takashi Matsunaga, Naohiko Okamoto, Sadao Imao

**Affiliations:** ^1^Department of Urology, Kanto Rosai Hospital, 1-1 Kizukisumiyoshi-cho, Nakahara-ku, Kawasaki 211-8510, Japan; ^2^Department of Hematology, Kanto Rosai Hospital, 1-1 Kizukisumiyoshi-cho, Nakahara-ku, Kawasaki 211-8510, Japan

## Abstract

Hemorrhagic cystitis is a disorder which causes bleeding from diffusely inflammatory bladder mucosa. Here we present a case of severe hemorrhagic cystitis caused by melphalan. A 70-year-old man with multiple myeloma was presented with suddenly commenced massive gross hematuria. During an attempt of transurethral coagulation of bladder mucosa, bladder perforation into peritoneal cavity was suspected, then open laparotomy was indicated. We isolated bilateral internal iliac arteries and ligated them in order to control continued bleeding. After that, bladder bleeding was suddenly diminished. Ligation of internal iliac arteries may be a choice in controlling massive bleeding from bladder with severe hemorrhagic cystitis when laparotomy was inevitable.

## 1. Introduction

Hemorrhagic cystitis is a disorder which causes bleeding from diffusely inflammatory bladder mucosa. Etiology [1] is chemicals such as cyclophosphamide, ifosphamide, and methotraxate, previous irradiation to pelvic organs [2], viral infection to the bladder notably by adenovirus BK virus especially in bone marrow, and stem cell transplant in patients with hematologic disorders [3]. 

As conservative therapies of hemorrhagic cystitis [1], bladder irrigation with saline or alkalinized saline, intravesical instillation of aluminum/hydroxide/magnesium hydroxide (Maalox) [4], hyperbaric oxygen therapy, and pain control are performed. Severe hemorrhagic cystitis is sometimes life-threatening. More aggressive therapy including transurethral coagulation of bladder (TUC), bladder furgulation or fixation with silver nitrate and formalin, transarterial embolization of internal iliac arteries or their branches to bladder [5], and finally cystectomy with urinary diversion [6] are reported. In a retrospective study, three out of 1300 bone marrow allograft recipients needed subtotal cystectomy with ileocystoplasty due to life-threatening hemorrhagic cystitis [7]. Here we present a case of severe hemorrhagic cystitis which caused bladder perforation during TUC for massive bladder bleeding, leading to laparotomy and the ligation of bilateral internal iliac arteries for hemostasis.

## 2. Case Presentation

A 70-year-old man with multiple myeloma was presented with suddenly commenced massive gross hematuria to Kanto Rosai Hospital. He had been administered melphalan (10 mg/day for four days) together with predonisolone (70 mg/day for four days) and zoledronic acid (4 mg on day 1) every four weeks as a treatment of multiple myeloma before. Eleven courses of that treatment were given in total. After admission, he underwent continuous bladder irrigation with saline and occassional evacuation of blood clot, nevertheless anemia and bladder tamponade progressed. Hemoglobin level dropped from 11.8 g/dl to 4.7 in the course of bladder bleeding. Adenovirus, BK virus, and herpes simplex virus were not detected in urine by the PCR amplification method.

**Figure 1:**
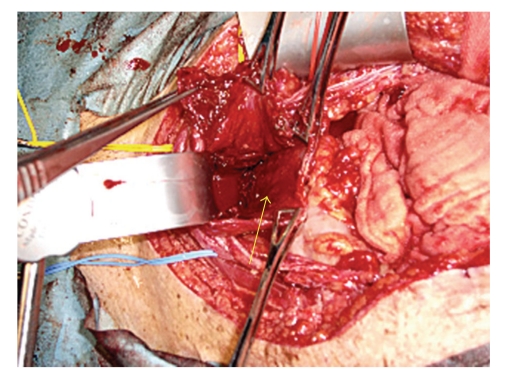
Intraoperative photograph shows inflammatory bleeding bladder mucosa. An arrow indicates incised bladder.

During transurethral coagulation of bladder mucosa, his abdomen begun to distend and bladder perforation into peritoneal cavity was suspected, then open laparotomy was indicated. After cystotomy, perforation of 10 mm in length was easily found at the bladder doom and repaired with absorbable sutures. Bleeding from wide area of the bladder (Figure 1) did not cease by the compression of bladder mucosa with gauze, then we isolated bilateral internal iliac arteries and ligated them with 1-0 silk. After that, bladder bleeding was suddenly diminished, although very slight macrohematuria continued thereafter. Cystostomy was created and continuous bladder irrigation utilizing both cystostomy and 3-way urethral catheter was performed without trouble thereafter.

**Figure 2:**
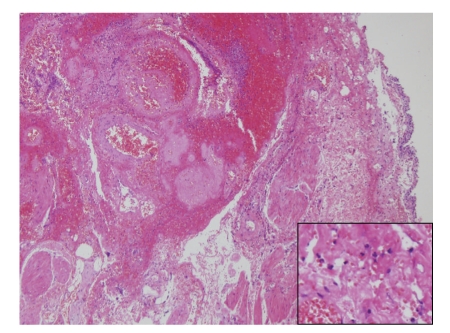
Histology of bladder specimen excised during laparotomy showing edema, bleeding, and granulocyte infiltration.

Histology of bladder specimen excised at laparotomy showed prominent edema and bleeding with granulocyte infiltration as in Figure 2. From the 17th day following arterial ligation, 50 ml of aluminum hydroxide gel (Malfa suspension) was intravesically instillated followed by a catheter clamp for one hour. After 21 days following arterial ligation, macroscopic hematuria completely disappeared. Total transfusion in the course was 26 units of red cells concentrates—leukocytes reduced, 20 units of platelet concentrates, and 5 units of fresh frozen plasma. Transfused red cells concentrates were 18 units pre- and intraoperatively and 8 units postoperatively, the latter was mainly to supplement pre-operative blood loss.

## 3. Discussion

Melphalan is supposed to be causative of hemorrhagic cystitis in this case, because there are no other antitumor drugs administered, no irradiation history, and no viruses detected in urine. Hemorrhagic cystitis is reported in cases where melphalan was used in combine with other hemorrhagic cystitis—inducing treatments like cyclophosphamide and irradiation [8–10]. But there is no report of hemorrhagic cystitis which was definitely brought about by melphalan. This case may be the first to show direct linkage of melphalan administration and severe hemorrhagic hematuria as a consequence.

We could preserve bladder by controlling massive bleeding with ligation of bilateral internal iliac arteries, which could avoid cystectomy. Transarterial embolization of internal iliac arteries or their branches could have controlled bladder bleeding if it had been tried instead of TUC, but laparotomy was inevitable in this case as bladder perforation into peritoneal cavity occurred during TUC possibly due to elevated intravesical pressure following bladder irrigation for acquiring visualization of the operative field and an attempt to evacuate massive blood clot in the bladder. Additionally, shutdown of bloodstream to the bladder may be more complete by ligating internal iliac arteries than by transarterial embolization, resulting in better hemostasis.

Cystectomy with urinary diversion might have been an appropriate choice. But we were reluctant to remove bladder without a fully informed consent to the patient. Moreover, cystectomy could have a possibility to induce serious complications if performed for a seriously ill patient with severe anemia. In conclusion, ligation of internal iliac arteries may be a choice in controlling massive bleeding from bladder with severe hemorrhagic cystitis when laparotomy was inevitable.
